# Flexible Transparent Electrodes Based on Gold Nanomeshes

**DOI:** 10.1186/s11671-019-2973-3

**Published:** 2019-04-16

**Authors:** Zeping Li, Geng Wang, Zhongming Li, Zhengze Cheng, Guopeng Zhou, Shan Li

**Affiliations:** 1 School of Electronic Information and Engineering, Hubei University of Science and Technology, Xianning, 437005 Hubei People’s Republic of China; 2 School of Petrochemical Engineering, Changzhou University, Changzhou, 213164 Jiangsu People’s Republic of China

**Keywords:** AuNM, NSL, FEA, Transmittance, Sheet resistance, Flexibility

## Abstract

The transmittance, conductivity, and flexibility are the crucial properties for the development of next-generation flexible electrodes. Achieving a good trade-off between transmittance and conductivity of flexible electrodes has been a challenge because the two properties are inversely proportional. Herein, we reveal a good trade-off between transmittance and conductivity of gold nanomesh (AuNM) can be achieved through appropriately increasing the AuNM thickness no more than 40 nm, the mean free path of electrons in Au metal. The further flexibility investigation indicates that the AuNM electrodes with mesh structure show higher tolerance than the Au bulk film, and the AuNM electrodes with smaller inter-aperture wire width can accommodate more tensile strains than a counterpart with bigger inter-aperture wire width. The simulated results based on finite element analysis (FEA) show good agreement with experimental results, which indicates the fabrication method of versatile nanosphere lithography (NSL) is reliable. These results established a promising approach toward next-generation large-scale flexible transparent AuNM electrodes for flexible electronics.

**Electronic supplementary material:**

The online version of this article (10.1186/s11671-019-2973-3) contains supplementary material, which is available to authorized users.

## Introduction

Recently, novel flexible transparent electrodes have been investigated, such as doped metal oxides (ITO, FTO), carbon nanotubes, graphene, and conducting polymers, to enable electrical conductivity and optical transparency simultaneously under mechanical deformation. [1–5]. ITO and FTO suffer from manufacturing cost and brittleness due to their ceramic nature, which confines the application on irregular surfaces [6, 7]. The poor environmental stability and biocompatibility of conducting polymers by reason of the instability of the doped state have been unresolved [8]. One primary strategy is to use highly conductive metal nanomesh materials on an elastic substrate [9]. The metal film as transparent electrodes stems largely from their typically high free-electron density, which enables ultrathin metal film on the order of 1–40 nm thickness to have optical transparency and appropriate conductivity [10]. However, a single ultrathin metal film cannot have high transmittance because of the high surface reflection even if absorption inside the metal film is negligible by setting its thickness comparable to the skin depth [11, 12]. To address the issues, the nanostructured transparent metal electrodes have been recently developed to allow the light to pass through and conceivably achieve high optical transmission while maintaining the low sheet resistance of the metal and effective flexibility [13–17]. Silver nanowire showed low sheet resistance and high transparency as flexible transparent electrodes for replacing ITO [13–15]. However, several drawbacks, such as large junction resistance, small contact area, and easy corrosion due to oxidation and sulfur vulcanization, degraded the performance of the silver nanowire electrodes [10]. Considering the issue of long-term stability, some metals such as Au and Pt should be developed firstly, by virtue of their long-term electrical stability without corroding through oxidation [16, 17]. The transparent AuNM electrodes with mesh-like topology have been increasingly explored for better performance [18, 19]. However, achieving a good trade-off between transmittance and conductivity of AuNM has been a challenge because the two properties are inversely proportional [20, 21]. The influence of mesh size on mechanical flexibility properties has not been investigated for them to be applied to flexible electronics [22].

In this paper, we demonstrate the flexible transparent AuNM electrodes made by the versatile nanosphere lithography (NSL) technique [23–25]. The resulting AuNM electrode with hexagonal, uniform, and periodic nanostructure exhibited excellent transmittance and sheet resistance. The simulated results based on finite element analysis (FEA) show good agreement with experimental results, and the results reveal a good trade-off between transmittance and conductivity of AuNM can be achieved through appropriately increasing the AuNM thickness no more than 40 nm. The further flexibility investigation indicates that the AuNM electrodes with mesh structure show higher tolerance than the Au bulk film, and the AuNM electrodes with smaller inter-aperture wire width can accommodate more tensile strains than a counterpart with bigger inter-aperture wire width. The bench tests indicate the prepared AuNM electrodes possess high transmittance, low sheet resistance, and excellent flexibility.

## Methods and Experiments

### Experimental Details

NSL attracts more and more attention as an inexpensive and wafer-scale technique for the fabrication of ordered, uniform, and tunable nanostructure utilizing a hexagonally close-packed monolayer of polystyrene spheres (PS, Aladdin Co., Ltd.) as a template [26–28].

**Fig. 1:**
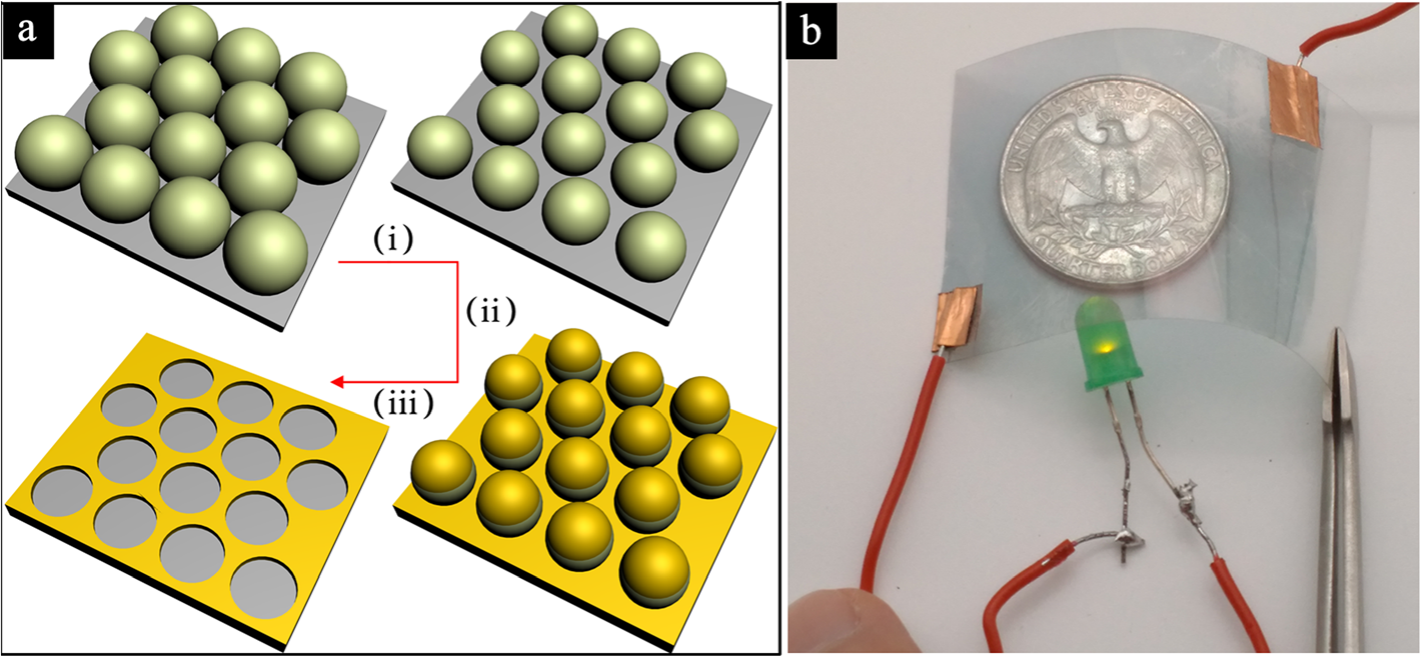
**a** The preparation flow schematic of AuNM electrode. **b** The demonstration of transmittance and conductivity performance

Figure 1a shows the fabrication process for AuNM using the NSL technique. (i) After a close-packed monolayer of PS spheres with an initial diameter *D* = 1 μm was deposited onto a 500-μm-thick polyethylene terephthalate (PET, Aladdin Co., Ltd.) substrate on glass, which was cleaned with isopropanol and deionized water sequentially via an air/water interface with self-assembly, the diameter of the PS spheres was reduced via reactive ion etching (RIE, etching gases: O_2_ and CHF_3_) to create gaps between the PS spheres. (ii) Metal nanomesh was formed in the vacancies between the PS spheres after the deposition of 2 nm Ti buffer layer and 20 nm Au via electron beam evaporation. (iii) After PS spheres were removed by an adhesive tape and sonication, the metal nanomesh on substrate was yielded. The obtained microstructures were characterized by a scanning electron microscope (SEM, Nova NanoSEM 450, FEI, Eindhoven, Netherlands). To visually demonstrate the performance of transmittance and sheet resistance under strain tension, we developed a measurement setup as shown in Fig. 1b. In this test, a typical AuNM membrane with ~ 160 nm average inter-aperture wire width and ~ 20 nm thickness on PET film (thickness ~ 500 μm) was adopted. The transparent and bent AuNM electrode under strain tension is connected with the wire by conductive silver paste and conductive copper tape for good electrical contact, yielding lit of LED, as shown in Fig. 1b. This test indicates the prepared AuNM electrodes possess high transmittance, low sheet resistance, and excellent flexibility.

**Fig. 2:**
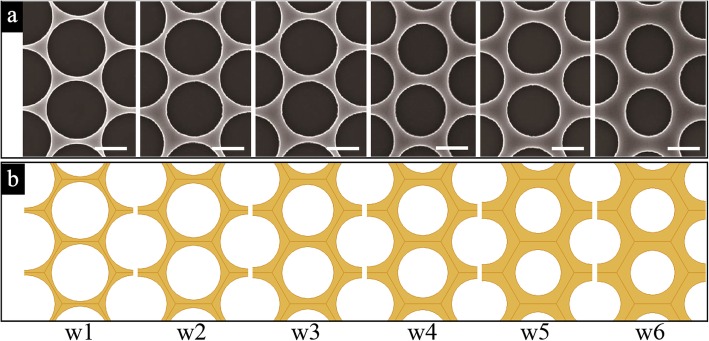
The structure images of AuNM. **a** Top-view SEM images of six different experimental samples, and **b** top-view drawings of six different numerical models. Scale bar: 500 nm

As shown in Fig. 2a, the prepared AuNM has a precisely controlled nanostructure showing excellent uniformity with hexagonally arrayed periodic circular holes. The six different AuNM samples with averaged inter-aperture wire width, namely the vacancies between two PS spheres (labeled as “w,” varying from 100 nm to 175 nm, w1 = 100 nm, w2 = 115 nm, w3 = 130 nm, w4 = 145 nm, w5 = 160 nm, w6 = 175 nm), were prepared for comparison.

### Simulation Details

For comparison, the six different numerical models (Fig. 2b) with the same parameters as the prepared AuNM samples have been analyzed in the FEA simulation.

In the electromagnetic simulations, the light source was set to create circularly polarized light on a unit cell of AuNM on PET, as shown in Additional file 1: Figure S1. An integrating sphere was used to measure the total transmitted light and not simply the specular transmittance. Periodic boundary conditions were used to simulate in one unit cell at the horizontal directions. And perfectly matched layer boundary conditions were used to prevent unphysical scattering at the edge of the simulated unit cell in the vertical directions [29]. Further, parameters of material properties were applied from the published experimental data, which were the same for the material of mechanic simulations [30]. Additional file 1: Figure S2 shows a schematic diagram of the models of AuNM and AuNM on PET in mechanical flexibility simulation, respectively.

## Results and Discussions

**Fig. 3:**
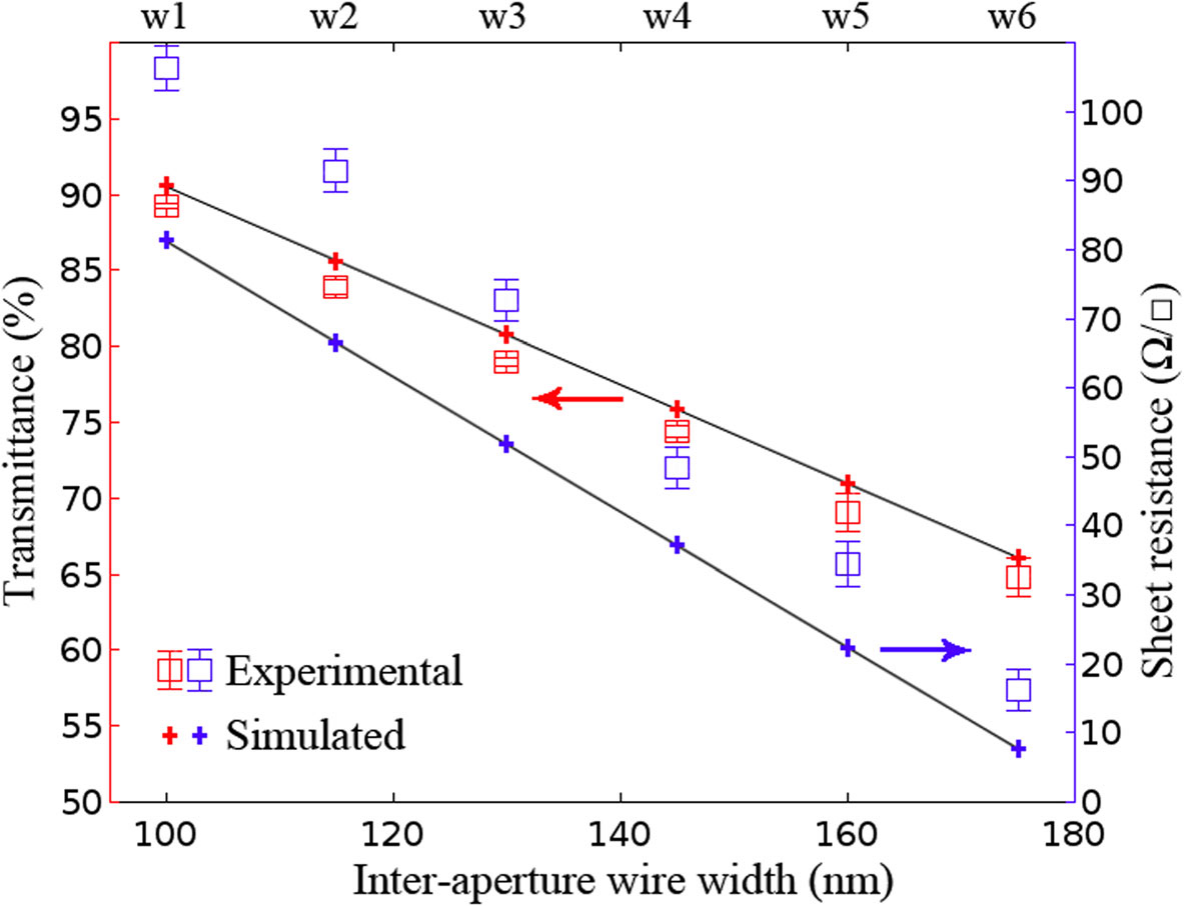
Transmittance and sheet resistance vs. inter-aperture wire width of AuNM (at *λ* = 550 nm and thickness = 20 nm)

The theoretical model is validated by comparing the simulated results with the experimental data. The transmittance at 550 nm and sheet resistance properties of six different samples based on the simulated and experimental data are demonstrated in Fig. 3. Along with the increase of inter-aperture wire width, both of the transmittance and the sheet resistance decreased. In particular, the variation trend of simulated data is linear. The measured transmittance and sheet resistance properties are in agreement with the simulated properties, which indicate the NSL fabrication method is reliable. The largest transmittance of 89% and sheet resistance of 104.5 Ω/□ were measured at the smallest wire width of 100 nm, and the largest wire width of 175 nm yields transmittance of 65% and sheet resistance of 16.5 Ω/□. From geometric considerations, higher transmittance derives from bigger apertures, namely smaller wire width due to the decrease of etching time for PS spheres, which results in a decreased area to block the light. However, smaller wire width results in increasing the sheet resistance due to decreased conducting pathways for electrons to flow.

It should be noted that the transmittance and sheet resistance decreased linearly as the inter-aperture wire width increased in simulated results is by virtue of perfect periodicity of simulated models. On the contrary, performances of transmittance and sheet resistance in the experimental results suffer from degradation due to more or less some inevitable defects, impurities, and surface roughness.

**Fig. 4:**
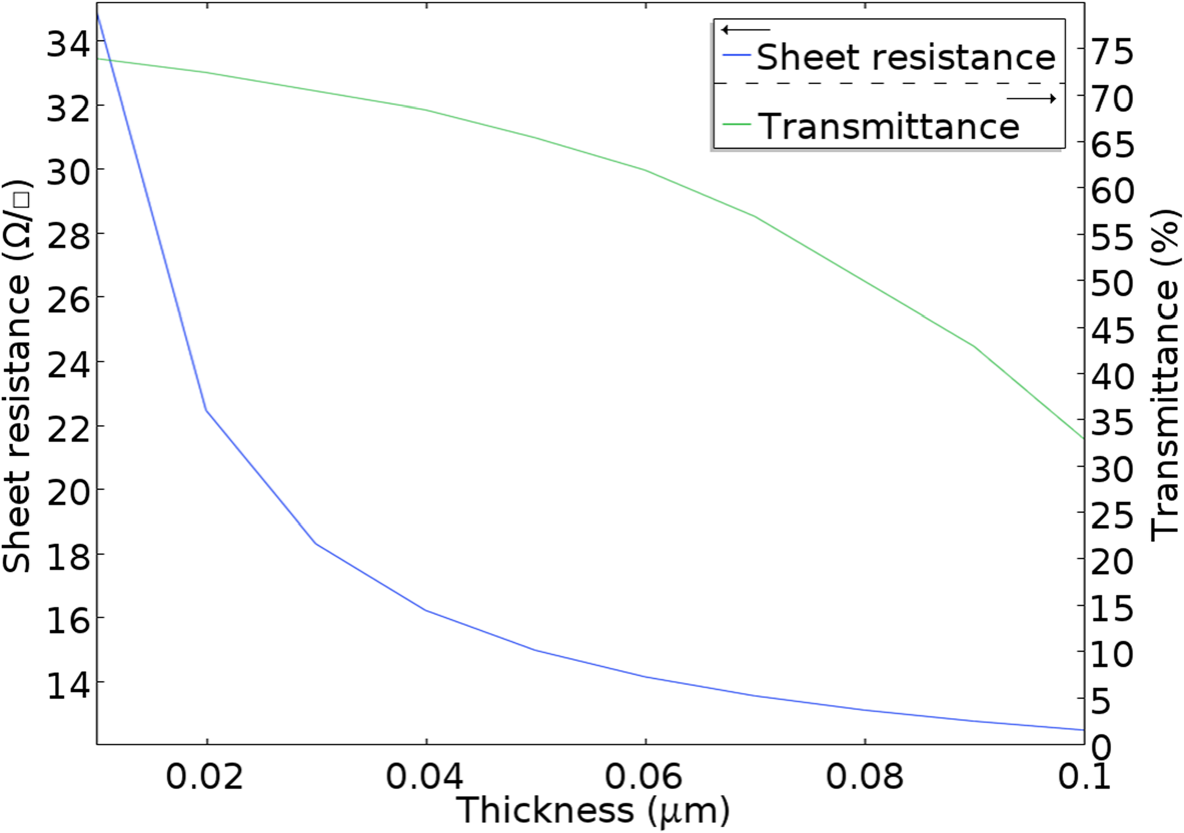
Transmittance and sheet resistance vs. the AuNM thickness (at *λ*= 550 nm and W5 = 160 nm)

In order to maximize the potential of AuNM for use as a transparent electrode, it is typically desirable to have a high transmission and a low sheet resistance. However, achieving a good trade-off between transmittance and conductivity of AuNM has been a challenge because the two properties are inversely proportional. To address the issue, herein, we analyzed theoretically the effect of AuNM thickness on transmittance and sheet resistance. All the simulations were performed at the same 550 nm wavelength, 160 nm averaged inter-aperture wire width, and 10 to 100 nm thickness. Additional file 1: Figure S3 shows the potential distribution map of AuNM at constant current. At the initial stage in Fig. 4, the increase of AuNM thickness results in a rapid decrease of sheet resistance, which decreases slowly after the thickness of 40 nm. The thicker AuNM beyond 40 nm close to the mean free path of electrons in Au metal cannot significantly increase the conductivity [31]. Meanwhile, high transmittance has been maintained for a long time, which decreases slowly. Thicker AuNM would increase conducting pathways for electrons to flow, which yields a low sheet resistance with a slight degradation of transmission due to constant apertures and wire width.

Efforts could be dedicated to improve the transmittance and conductivity of such metal nanomesh by appropriately increasing the AuNM thickness no more than 40 nm, the mean free path of electrons in Au metal.

**Fig. 5:**
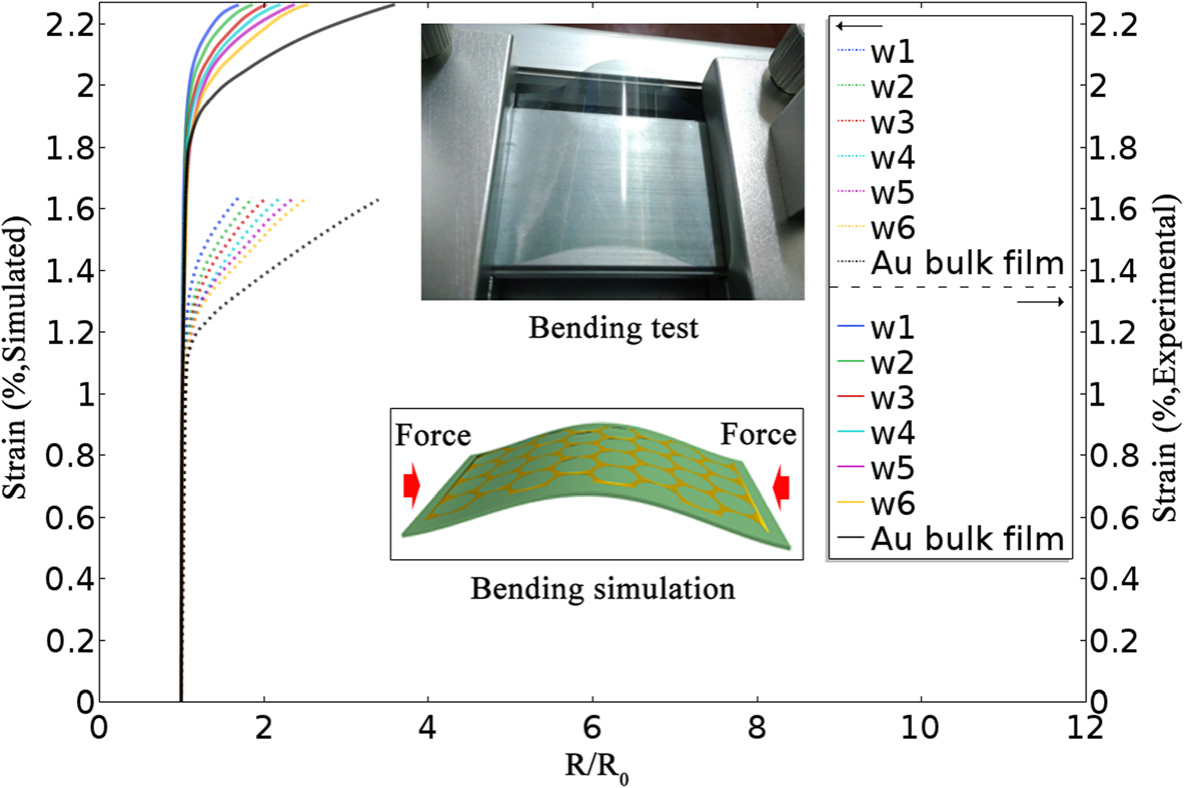
The strain level vs. *R*/*R*_0_ for AuNM electrodes and Au bulk film (*R*/*R*_0_, where *R*_0_ is the initial resistance under zero strain). The insets show maps of the AuNM electrodes during bending test and bending simulation, respectively

One compelling property of AuNM is good mechanical flexibility. The influence of strain on the sheet resistance was investigated to examine the mechanical flexibility of the AuNM under bending. In order to facilitate analysis, a sample of Au bulk film with the same parameters as a model counterpart of numerical Au bulk film (thickness ~ 20 nm) on PET film (thickness ~ 500 μm) has been fabricated. The insets show maps of the AuNM electrodes during bending test and bending simulation, respectively. Additional file 1: Figure S4 shows the stress distribution map of the AuNM electrodes during bending simulation under 1.5 × 10^9^ N/m^2^ force at the *Y* direction, which shows that stress is mainly concentrated in the center of AuNM. As shown as Fig. 5, in the bending test, firstly, the Au bulk film with maximum inter-aperture wire width exhibited a dramatic increase of the sheet resistance at strain beyond 1.9 % and the worst flexible performance. However, six AuNM electrodes remained their initial resistance until the stretch ratio reaches 2.1 %. At the same time, as inter-aperture wire widths decreased, the AuNM electrodes suffer from electrical failure gradually, due to the breakdown of the AuNM electrodes entirely.

It is not hard to find the AuNM electrodes with a mesh structure showing higher tolerance than the Au bulk film, and the AuNM electrodes with smaller inter-aperture wire width exhibit better flexible performance. The applied force on samples will cause tensile strain, which can be accommodated by in-plane rotations and distortion of periodic nanomesh without breakage of AuNM [32]. However, Au bulk film cannot accommodate the applied tensile strains, which cause its breakage at the threshold point of tensile strains and electrical failure.

The simulated results show good agreement with experimental results except that the threshold point of tensile strains in the simulated results (closed to 1.2) is lower than the experimental results. This is due to the fabricated samples with a size of several square centimeters can accommodate more tensile strains than the simulated models with a size of several square microns.

**Fig. 6:**
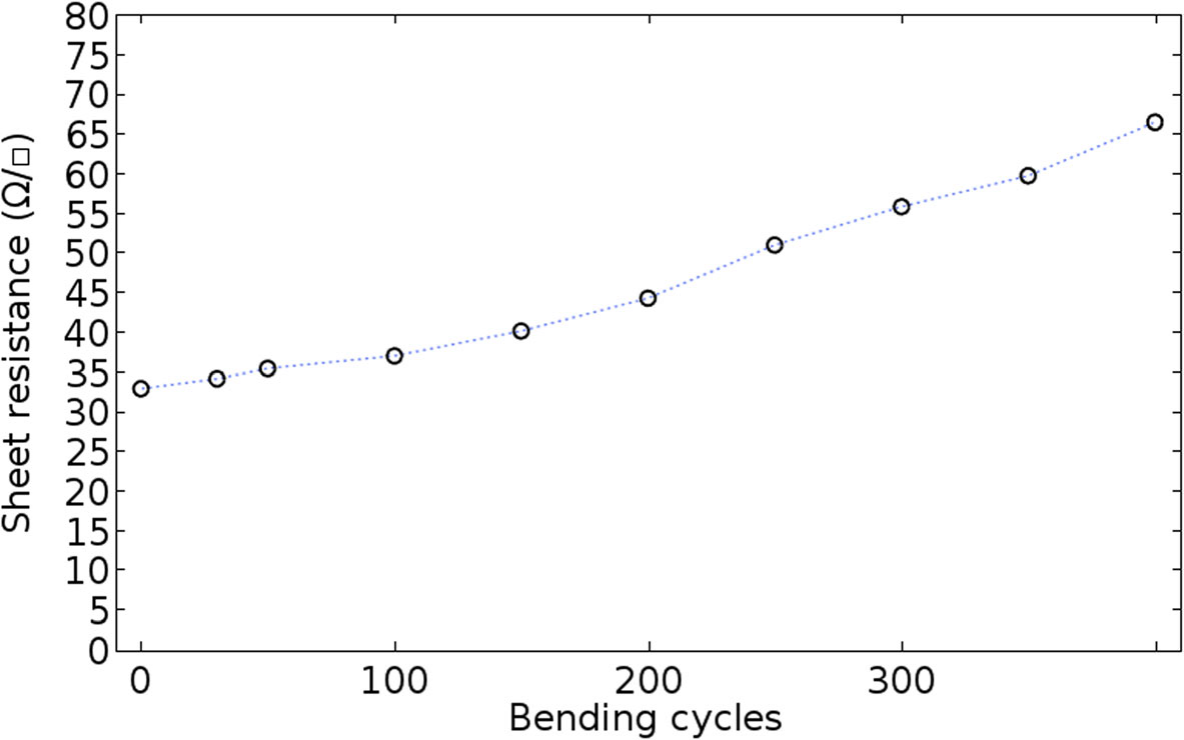
The sheet resistance vs. bending cycles in the mechanical bending test of AuNM (at W5 = 160 nm and thickness = 20 nm)

In addition, to assess the electrode stability, the sheet resistance value of the AuNM electrodes was measured as bending test progressed. AuNM electrodes on PET film were bent up to 400 cycles under a minimum radius of curvature of 5 mm and maximum of 15 mm, as shown in Fig. 6, showing the good flexible stability.

### Conclusions

In conclusion, the present results show the flexible transparent AuNM electrodes can be synthesized using the versatile NSL technique. The resulting AuNM electrode with hexagonal, uniform, and periodic nanostructure exhibited excellent transmittance and sheet resistance. The simulated results show good agreement with experimental results, which indicate the NSL fabrication method is reliable. A good trade-off between transmittance and conductivity of AuNM can be achieved through appropriately increasing the AuNM thickness no more than 40 nm, the mean free path of electrons in Au metal. In the flexibility investigation, the AuNM electrodes with mesh structure show higher tolerance than the Au bulk film, and the AuNM electrodes with smaller inter-aperture wire width can accommodate more tensile strains than a counterpart with bigger inter-aperture wire width; the mechanical bending test shows the good flexible stability of AuNM. The prepared AuNM electrodes with high transmittance, low sheet resistance, and excellent flexibility established a promising approach toward next-generation large-scale flexible transparent AuNM electrodes, with broad utility for applications in flexible electronics including biosensors and optoelectronic devices.

## Additional file

Additional file 1: **Figure S1.** Schematic diagram of the simulated unit cell of AuNM on PET. (a) Top view. (b) Perspective view. **Figure S2.** Schematic diagram of the simulated models. (a) AuNM. (b) AuNM on PET. **Figure S3.** The potential distribution map of AuNM at constant current. **Figure S4.** The stress distribution map of the AuNM electrodes during bending simulation under 1.5 × 10^9^N/m^2^ force at the *Y* direction. (DOC 1287 kb)

## Abbreviations

AuNM: Gold nanomesh
FEA: Finite element analysis
NSL: Nanosphere lithography
PET: Polyethylene terephthalate
PS: Polystyrene spheres
SEM: Scanning electron microscope
