# Supplementary material for: Flexible Transparent Electrodes Based on Gold Nanomeshes
Source: Nanoscale Res Lett. 2019 Apr 16;14:132. doi: 10.1186/s11671-019-2973-3 (PMC6468033; doi:10.1186/s11671-019-2973-3)
Supplement: Supplementary file 1 — Figure S1. Schematic diagram of the simulated unit cell of AuNM on PET. (a) Top view. (b) Perspective view. Figure S2. Schematic diagram of the simulated models. (a) AuNM. (b) AuNM on PET. Figure S3. The potential distribution map of AuNM at constant current. Figure S4. The stress distribution map of the AuNM electrodes during bending simulation under 1.5 × 10^9^N/m2 force at the Y direction. (DOC 1287 kb) [file 11671_2019_2973_MOESM1_ESM.doc]

# **Supplementary Information**

# **Flexible transparent electrode based on gold nanomeshes**

## Zeping Li^1*^, Geng Wang^1^, Zhongming Li^1^, Zhengze Cheng^1^, Guopeng Zhou^1^, Shan Li^2^

1 School of Electronic Information and Engineering, Hubei University of Science and Technology, Xianning, Hubei 437005, P.R. China

2 School of Petrochemical Engineering, Changzhou University, Changzhou, Jiangsu 213164, P.R. China

***To whom correspondence should be addressed.**

E-mail: D201477516@alumni.hust.edu.cn


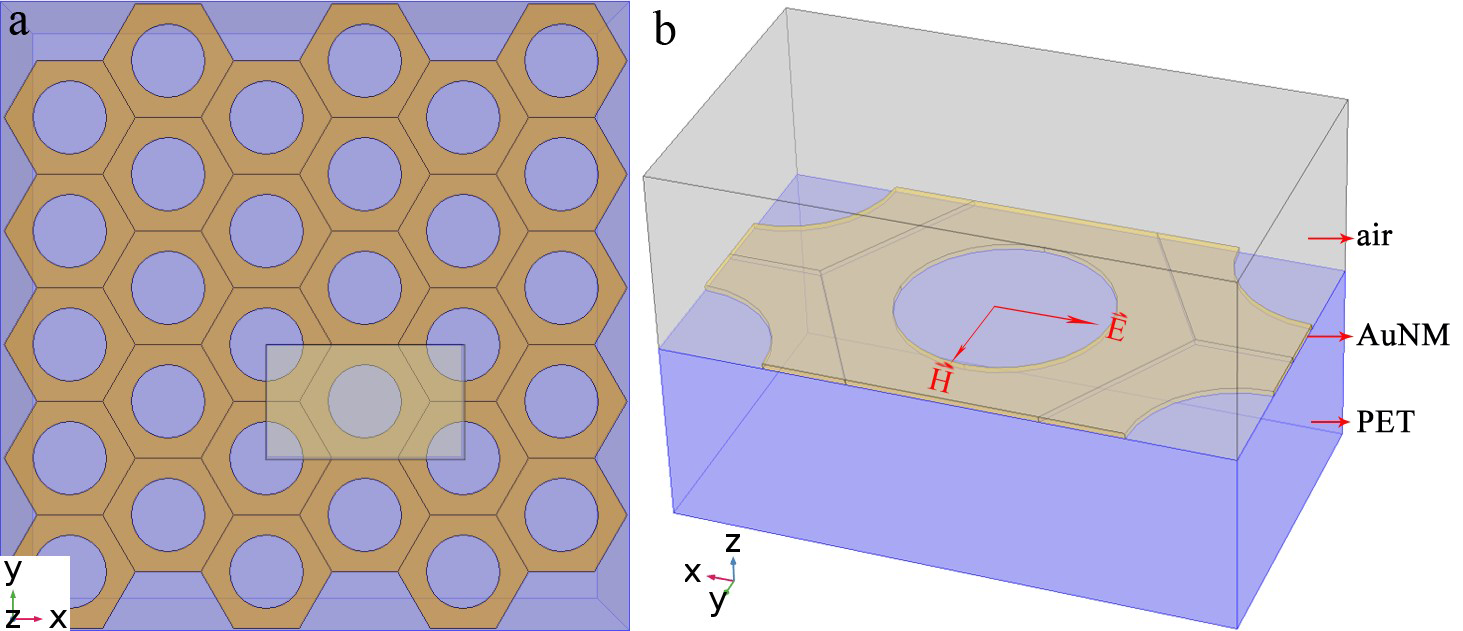


Supplementary **Fig. S1** Schematic diagram of the simulated unit cell of AuNM on PET. (a) Top view, (b) Perspective view.


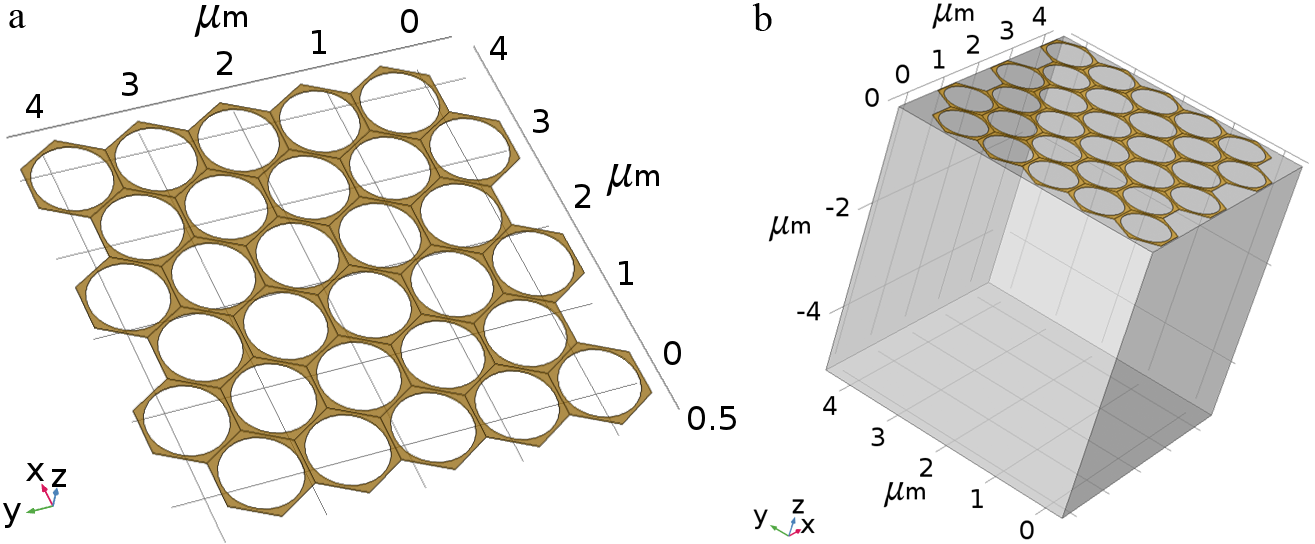


Supplementary **Fig. S2** Schematic diagram of the simulated models. (a) AuNM, (b) AuNM on PET.


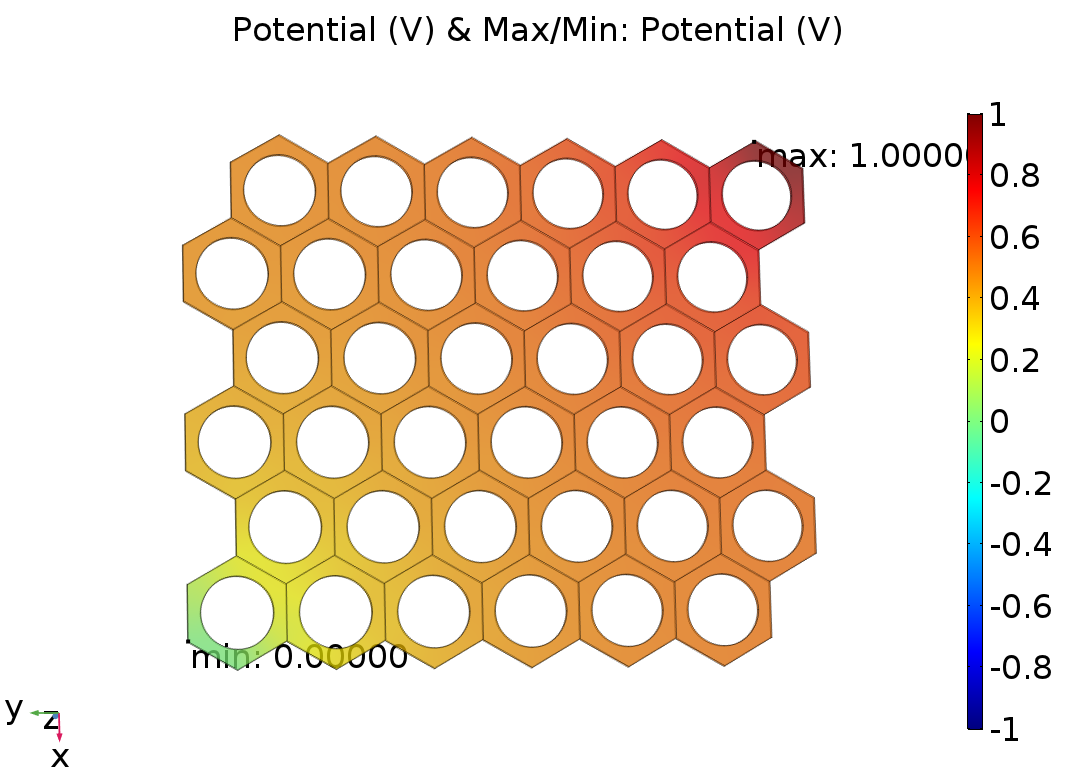
Supplementary **Fig. S3** The potential distribution map of AuNM at constant-current.


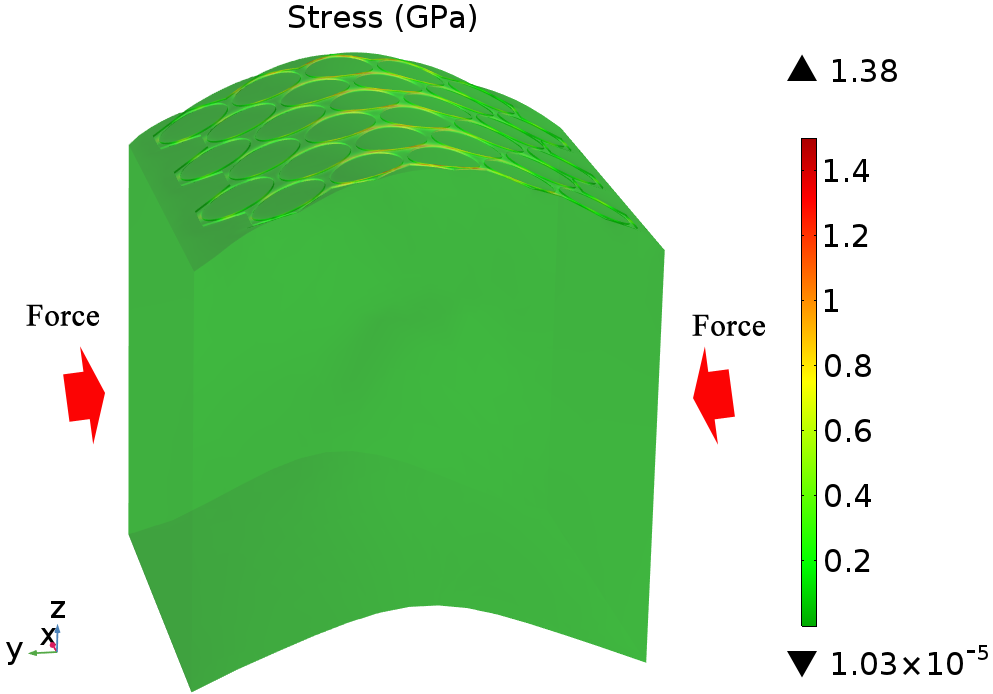


Supplementary **Fig. S4** The stress distribution map of the AuNM electrodes during bending simulation under 1.5*10^9^N/m^2^ force at the Y direction.

**References**

[1] Qiang Y, Seo KJ, Zhao XY, et al. (2017) Bilayer nanomesh structures for transparent recording and stimulating microelectrodes. Adv Funct Mater 27:1704117

[2] Winton SC, Kosmas P, Rappaport CM (2005) FDTD simulation of TE and TM plane waves at nonzero incidence in arbitrary layered media. IEEE T Antenn Propag 53:8418549

[3] Vayron R, Nguyen VH, Bos R (2015) Finite element simulation of ultrasonic wave propagation in a dental implant for biomechanical stability assessment. Biomech Model Mechan 14:1021-1032

[4] Matsuzaki R, Kawasaki M, Todoroki A (2015) Uncertainty Visualization of Estimated Damage Using Kriging Model: Application to Time-Domain Reflectometry Sensing. J Nondestruct Eval 34:23
